# Comparison of regional and local anesthesia for arteriovenous fistula creation in end-stage renal disease: a systematic review and meta-analysis

**DOI:** 10.1186/s12871-020-01136-1

**Published:** 2020-08-31

**Authors:** Chen Gao, Chunyan Weng, Chenghai He, Jingli Xu, Liqiang Yu

**Affiliations:** 1 Department of Nephrology, The Hangzhou Fuyang Hospital of Traditional Chinese Medicine, Zhejiang, Hangzhou China; 2 The First Clinical Medical of Zhejiang Chinese Medicine University, Zhejiang, Hangzhou China; 3 Department of Internal Medicine, The Affiliated Hospital of Hangzhou Normal University, 126 Wenzhou Road, Zhejiang, Hangzhou China

**Keywords:** Arteriovenous fistula, End-stage renal disease, Local anesthesia, Regional anesthesia, Meta-analysis, Systematic review

## Abstract

**Background:**

Arteriovenous fistulae (AVF) are the hemodialysis access modality of choice for patients with end-stage renal disease. However, they have a high early failure rate. Good vascular access is essential to manage long-term hemodialytic treatment, but some anesthesia techniques directly affect venous diameter as well as intra- and post-operative blood flow. The main purpose of this meta-analysis was to compare the results of regional and local anesthesia (RA and LA) for arteriovenous fistula creation in end-stage renal disease.

**Methods:**

We conducted a systematic review and meta-analysis to synthesize evidence from 7 randomized controlled trials (565 patients) and 1 observational study (408 patients) with the aim of evaluating the safety and efficacy of RA versus LA in surgical construction of AVF.

**Results:**

Pooled data showed that RA was associated with higher primary patency rates than LA (odds ratio [OR], 1.88; 95% confidence interval [CI]: 1.24–2.84; *P* = 0.003; I^2^ = 31%). Additionally, brachial artery diameter was significantly increased in the RA versus LA group (mean difference [MD], 0.83; 95% CI: 0.75–0.92; *P* < 0.001; I^2^ = 97%) and the need for intra- as well as post-operative pain killers was significantly less (RA, *P* = 0.0363; LA, *P* = 0.0318). Moreover, operation duration was significantly reduced using RA versus LA (MD, − 29.63; 95% CI: − 32.78 - -26.48; *P* < 0.001; I^2^ = 100%).

**Conclusions:**

This meta-analysis suggests that RA is preferable to LA in patients with end-stage renal disease in guaranteeing AVF patency and increasing brachial artery diameter.

## Background

The construction of arteriovenous fistulae (AVF) is an established form of therapy for patients with chronic renal failure. However, the primary failure rate for AVF creation under local anesthesia (LA) for hemodialysis is very high; approximately one third of AVF fail at an early stage [1]. General anesthesia (GA), regional anesthesia (RA), and local anesthetic infiltration are three acceptable anesthetic techniques used for the surgical construction of AVF; however, the choice of anesthetic technique may significantly affect early patency or long-term AVF outcomes.

General anesthesia is associated with increased cardiorespiratory complications in patients with end-stage renal disease. Thus, in such patients, RA, such as a brachial plexus block (BPB), or LA are favored for AVF creation. However, whilst both local anesthetic infiltration and RA avoid the risks associated with GA, only RA may be used to produce an associated sympathetic nerve block, which increases venous diameter and arterial flow intraoperatively, as well as in the early postoperative period.

Compared with LA, BPB is thought to improve local hemodynamic parameters. However, the effects of both techniques on fistula patency and failure rates are highly controversial. Therefore, we conducted a systematic review and meta-analysis to collect evidence from published randomized controlled trials (RCTs) and observational studies to assess the safety and efficacy of LA and RA in the surgical creation of AVF.

## Methods

### Electronic searches

This systematic review and meta-analysis followed the Preferred Reporting Items for Systematic Reviews and Meta-Analyses (PRISMA) statement recommendations. We searched the literature using PubMed, EMBASE, and Cochrane library databases, and included studies published from August 1951 to September 2017. The Medical Subject Headings (MESH) search query used were as follows: arteriovenous fistula OR (arteriovenous AND fistula) AND (anesthesia OR local anesthesia OR brachial plexus anesthesia OR regional anesthesia OR anesthesia OR regional anesthesia OR brachial plexus block OR brachial plexus anesthesia OR brachial plexus blockade OR local anesthesia OR conduction anesthesia OR infiltration anesthesia). We also reviewed the reference lists of eligible studies and reviews to identify any additional relevant studies. Disagreement over relevance was resolved by consensus.

### Study selection

Study titles and abstracts were screened for eligibility by two independent reviewers. Eligible studies included open-label and double-blinded RCTs, as well as retrospective studies with adult open-label participants (≥ 18 years), that compared the efficacy of RA versus LA for AVF creation in end-stage renal disease. Studies meeting any of the following criteria were excluded: (a) animal-based studies; (b) studies not published in English; (c) abstracts, editorials, case reports, reviews, and case series.

The following data and outcomes were extracted and included in the study: (a) study characteristics (including: study design, sample size, follow-up duration, and publication year); (b) primary clinical outcomes (including: primary fistula patency rate, primary fistula failure rate, surgery duration, change in brachial artery diameter (mm), change in brachial artery blood flow rate (mL/min), and post-surgery comorbidities).

### Data analyses and quality assessment

We used Review Manager software (RevMan version 5.3) to analyze the extracted data. Odds ratios (ORs) were calculated with 95% confidence intervals (CIs). Heterogeneity between ORs for the same outcomes across different studies were explored using the I^2^ inconsistency test, which describes the percentage of total variation across studies due to heterogeneity as opposed to chance. A value of 0% indicates no observed statistical heterogeneity, whilst larger values signify more substantial heterogeneity.

The studies were assessed using the Cochrane risk of bias tool (Fig. 2) and the Newcastle-Ottawa Scale (Table 1). Disagreements between the two independent investigators were resolved via discussion.

**Table 1:** Risk of bias assessment

Study	Selection	Comparability	Exposure	Total score
Solomonson, et al. 1994	**	**	***	7

## Results

**Fig. 1:**
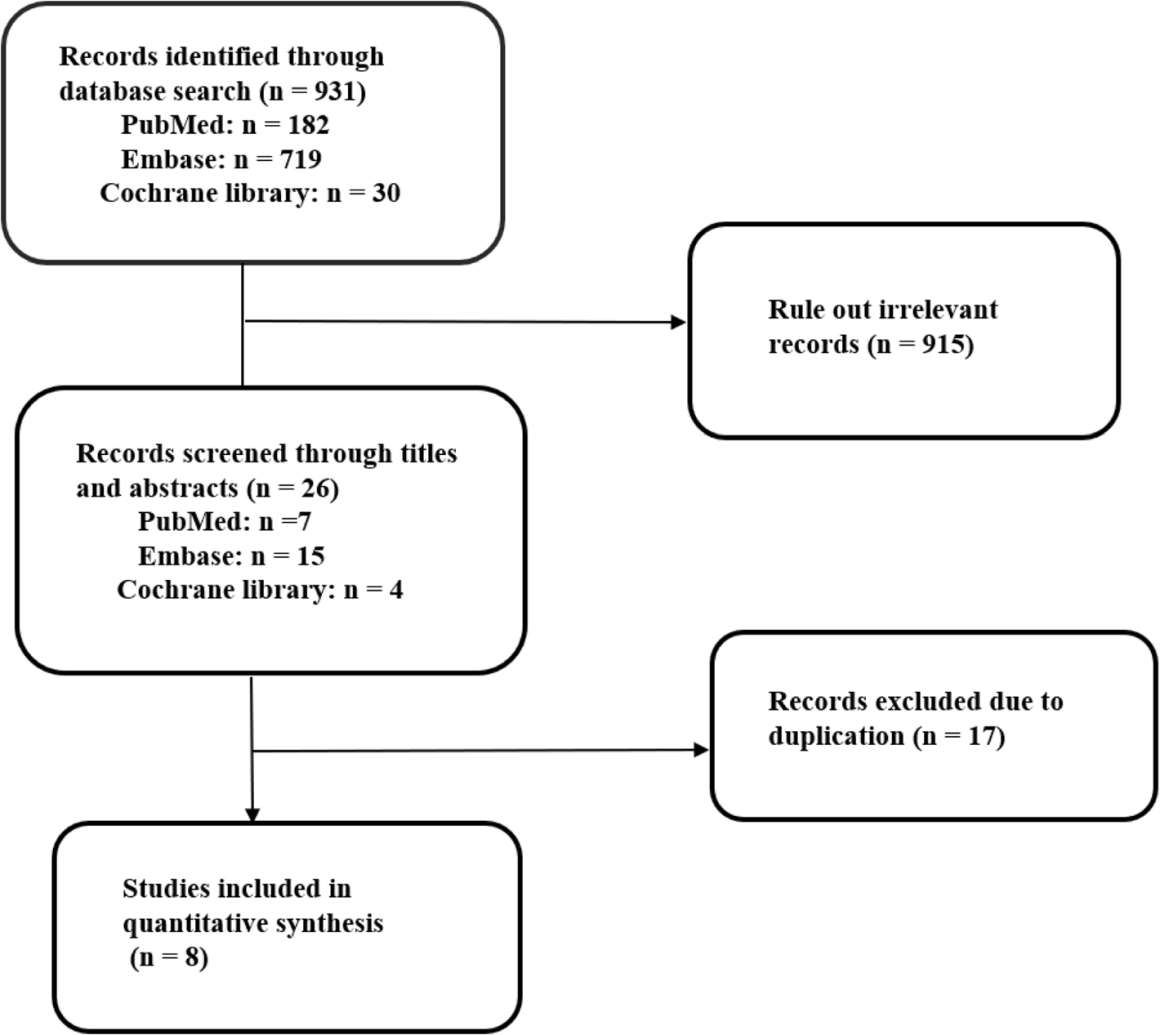
Study selection flow diagram

**Fig. 2:**
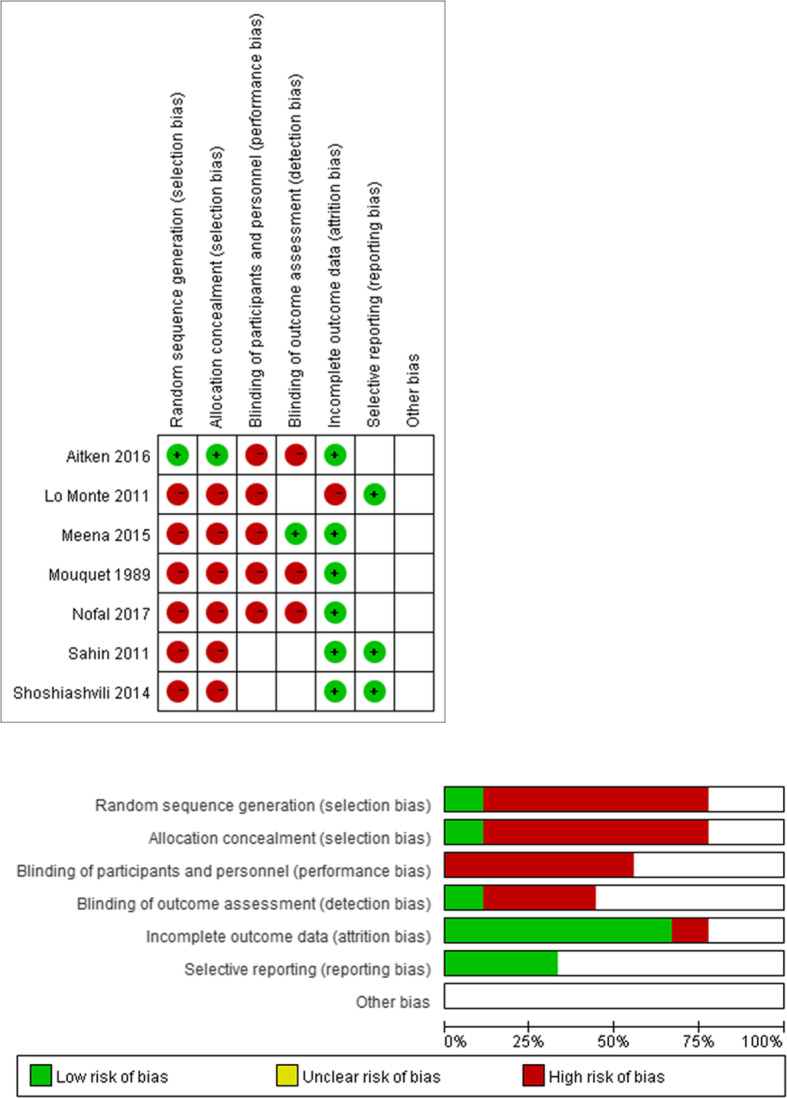
Risk of bias assessment

Details of the auto-selection process are outlined in Fig. 1. Overall, 8 studies, including 7 RCTs [2–8] and 1 retrospective study, [9] with a total of 955 patients, met the inclusion criteria. The characteristics of all included studies are provided in Table 2 Details of the quality assessments are provided in Fig. 2 and Table 1.

**Table 2:** Summary of included studies and baseline characteristics of their populations

Study	Design and study arms	Sample size (*n*)	Age (M ± SD, years)	Sex (*n*)	Comorbidities (*n*)	Duration of follow up	Outcomes Examined
Mouquet, et al. 1989	RCT (BPB vs. LA or GA)	18	52 ± 16	Male (23); Female (13)	–	2 h; 3 days; 10 days	Brachial artery blood flow
Solomonson, et al. 1994	Retrospective study (BPB vs. LA or GA)	408	63 ± 14	Male (245); Female (163)	Infection (16); Neuropathy (9); Seizure (1); Cardiac event (17)	–	Fistula failure; Graft infection, neuropathy in the extremity receiving the fistula; Seizure; Cardiac arrest; MI; Death within 7 days
Lo Monte, et al. 2011	RCT (BPB vs. LA)	40	BPB, 66.15 ± 7.55; LA, 66 ± 7.49	Male (23); Female (17)	Diabetes (15); High blood pressure (13); Systemic lupus erythematosus (5); Glomerulonephritis (4); Autoimmune vasculitis (3);	100 days	PI ratio; Venous / arterial diameter; Vein diameter
Sahin, et al. 2011	RCT (BPB vs. LA)	60	BPB, 43.4 ± 10.7; LA, 46.8 ± 12.5	Male (34); Female (26)	Diabetes (24); Hypertension (27); Coronary artery Disease (21)	3 h; 7 days; 8 weeks	Radial artery flow; Fistula flow; Thrill presence
Shoshiashvili, et al. 2014	RCT (BPB vs. LA)	103	BPB, 60.1 ± 14; LA, 59.7 ± 13	Male (68); Female (35)	Arterial hypertension (87); Diabetes (18); Ischemic heart disease (9); Gastric ulcer (1); Hepatitis B (2); Hepatitis C (7); Osteoblastoma (1)	100 days	Intra-operative pain; Need for intraoperative pain killers; Need for postoperative pain killers; Duration of anesthesia (h); Attitude to the type of anesthesia; Pain intensity, night sleep; Limb immobility; Operation duration (min)
Meena, et al. 2015	RCT (BPB vs. LA)	60	BPB, 41.33 ± 12.906; LA, 47.7 ± 12.272	Male (46); Female (14)	Diabetes (8); Hypertension (21); Hypertension (14); IgA (15)	30 min 48 h; 2 weeks; 6 weeks	Vessel diameter; Peak systolic velocity; Mean diastolic velocity; Blood flow
Aitken, et al. 2016	RCT (BPB vs. LA)	126	60.8 ± 14.8	Male (79); Female (47)	Diabetes (34); Ischemic heart disease (48); Cerebrovascular accident (9); Hypertension (93) Obesity (41)	3 months	Brachial artery blood flow; Radiocephalic fistulae; Cephalic vein (wrist) diameter (mm); Brachiocephalic fistulae; Brachial artery diameter (mm); Cephalic vein (elbow) diameter (mm)
Nofal, et al. 2017	RCT (BPB vs. LA)	140	BPB, 39.52 ± 5.46; LA, 42.42 ± 5.41	Male (79); Female (61)	–	4 h; 1 week; 3 months	Radial artery internal diameter; Cephalic vein internal diameter

*BPB* brachial plexus block, *IgA* immunoglobulin A, *GA* general anesthesia, *LA* local anesthesia, *MI* myocardial infarction, *PI* pulsatility Index Ratio, *RCT* randomized controlled trial, *M ± SD* mean ± standard deviatio

### Clinical outcomes

**Fig. 3:**
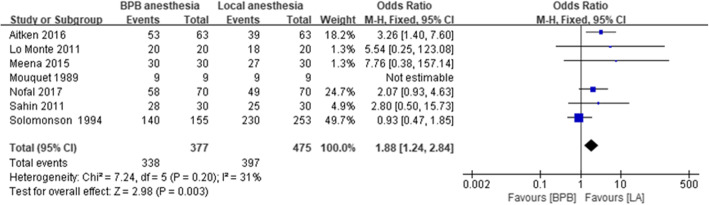
Patency of brachial plexus block (regional anesthesia) versus local anesthesia

In total, 7 studies, including 852 patients, [2, 3, 5–9] evaluated primary patency rates in RA versus LA; RA was associated with higher primary patency rates than LA (OR, 1.88; 95% CI: 1.24–2.84; *P* = 0.003; I^2^ = 31%; Fig. 3). The combined data from 3 trials, [6–8] including 284 patients, demonstrated that RA was associated with significantly increased brachial artery diameters compared to LA (mean difference (MD), 0.83; 95% CI: 0.75–0.92; *P* < 0.001; I^2^ = 97%). The combined data from 2 trials, [6, 8] including 144 patients, revealed that LA was associated with significantly reduced branchial artery blood flow compared to RA (MD, 47.5; 95% CI: 35.18–59.12; *P* < 0.001; I^2^ = 83%). Two trials, [4, 6] including 229 patients, reported data regarding operative times, demonstrating significantly longer operative times in RA versus LA (MD, − 29.63 min, 95% CI: − 32.78 - -26.48; *P* < 0.001; I^2^ = 100%). Details of the clinical outcomes are provided in Table 3.

**Table 3:** Main clinical results

Outcome variable	Number of convective therapy study arms	Number of patients	Absolute mean net change [95% CI]	I^2^
Duration of surgery	2	229	−29.63 [− 32.78, -26.48]	100%
Brachial artery diameter	3	284	0.83 [0.75, 0.92]	97%
Brachial artery blood flow rate	2	144	47.15 [35.18, 59.12]	83%
Complication of infection	3	594	0.68 [0.23, 2.02]	0%
Thrombosis	3	163	0.21 [0.03, 1.27]	0%
Hematoma	1	60	0.19 [0.01, 4.06]	–
Intraoperative analgesia	1	103	0.65 [0.30, 1.42]	–

*CI* confidence interval, *I*^*2*^ inconsistency test

### Complications

The combined data from 3 trials, [3, 6, 9] including 594 patients, demonstrated no difference between RA and LA in terms of vascular access infection (MD, 0.68; 95% CI: 0.23–2.02; *P* = 0.49; I^2^ = 0%). Three trials, [2, 3, 6] including 163 patients, revealed no significant difference between RA and LA with respect to the incidence of fistula thrombosis (OR, 0.21; 95% CI: 0.03–1.27; *P* = 0.09; I^2^ = 0%). Observations after BPBs in 1 trial, [3] including 60 patients, found no significant differences in the blocks until six-weeks post fistula creation (OR, 0.19; 95% CI: 0.01–4.06; *P* = 0.29; I^2^ = 0%). One trial, [4] including 103 patients, found a significant difference in pain intensity experienced between RA and LA (*P* = 0.0363 versus *P* = 0.0318, respectively), and time to postoperative pain initiation was significantly longer following RA versus LA. Operative duration was significantly shorter (*P* = 0.0007) for RA (67.5 ± 8.9 min) than LA (134.7 ± 14.8 min).

## Discussion

This meta-analysis included 955 patients from 8 studies (7 RCTs and 1 retrospective study). Combined data demonstrates that RA is associated with higher AVF primary patency rates and improved local blood flow compared with LA. Moreover, operation duration and the use of pain killers was significantly reduced with RA versus LA .

Axillary-approached BPB (RA) was preferable to LA. Arterial and venous dilation are crucial for AVF maturation [2] yet vascular surgery, such as local infiltration anesthesia, can easily lead to vessel spasm, impairing blood flow and potentially resulting in early fistula thrombosis. Comparatively, BPB can be performed using interscalene, supraclavicular, infraclavicular, and axillar approaches [4]. In a recent study, BPB was found to provide higher blood flow to the radial artery and AVF compared to infiltration anesthesia [3] given the sympatholytic effect, producing significant vasodilatation, decreased vascular resistance, [10] and increased local blood flow. This is consistent with other recent studies showing improvements in arterial blood flow and vasodilatation with RA. In a recent study by Nofal et al, [7] the overall mean AVF blood flow was 42.21 ml/min more in the BPB versus LA group. Similarly, a report by Malovrh [11] revealed a mean preoperative flow rate of 54.5 ml/min in BPB vessels with a successful outcome versus 24.1 ml/min in vessels that failed LA. In another study by Sahin et al, [3] improved blood flow in the radial artery was significantly greater post- versus pre-anesthesia. Moreover, post-anesthesia and immediately pre-surgery, radial artery blood flow was 56 ± 8.6 mL/min in the BPB group versus 40.7 ± 6.1 mL/min in the LA group (*P* < 0.001). Finally, Ebert et al [12] reported that both mean arterial and venous blood flow were increased (1.9 and 8.6 times, respectively) after BPB. Thus, we conclude that BPB anesthesia techniques in AVF construction can contribute to vessel dilation and reduced vasospasm via sympathectomy-like effects, increasing fistula blood flow, reducing fistula maturation time, and improving the success rates of vascular access procedures.

Arteriovenous fistulae operations can be performed under GA, LA, or RA. General anesthesia is associated with increased morbidity, [13] such as through cardiorespiratory complications in patients with end-stage renal disease, whilst LA is associated with complications such as vasospasm and pain and discomfort during surgery [10, 12, 14]. By comparison, RA (e.g. BPB), which is a targeted injection of LA to specifically block the motor and sensory nerves that supply the operative site, is less complicated than GA and safer than LA [15]. Moreover, BPB can be performed under ultrasound guidance, allowing for more accurate placement of the injection needle as well as more rapid onset and longer duration of the block, reduced vascular and neurological complications, and minimization of the volume of LA required [16, 17].

Pain control is also an important indicator of surgical success. Adequate pain control is extremely important in patients with end-stage renal disease with severe co-morbidities [15]. The prospective, randomized, clinical study from Shoshiashvili et al [4] showed significantly different results between BPB and LA groups in terms of pain intensity. The need for intra- as well as post-operative pain killers was significantly less in the BPB versus LA group (*P* = 0.0363 and *P* = 0.0318, respectively). Moreover, time to postoperative pain initiation was significantly higher in the RA versus LA group. Thus, we conclude that RA provides better pain control intra- as well as post-operatively in dialysis AVF operations, enabling patients to feel more comfortable [5].

The results of our study are consistent with those of previous meta-analyses. In a systematic review of 6 randomized trials (462 patients) and 1 retrospective study (408 patients), Ismail et al [18] reported that RA improves the primary patency rate of AVF compared to LA. In conclusion, our meta-analysis suggests that RA is preferable to LA in patients with end-stage renal disease in guaranteeing AVF patency and increasing brachial artery diameter.

## Limitations

Our study has several limitations. First, BPB can be performed with interscalene, supraclavicular, infraclavicular and axillar approaches. We included studies using different approaches for BPB, and did not consider the effects of these approaches in our comparison of LA versus RA. Future studies are thus required to explore the effect of different anesthetic approaches on the outcomes of BPB. Second, three of the studies included in this study were single-center trials with an inherent risk of bias. Moreover, there are relatively few primary studies available in the literature. Both factors restrict the generalizability of our findings. Third, only short-term data are reported in the literature; thus, future studies are required to explore longer-term outcomes. Finally, only one study explored patients’ attitudes towards anesthesia and, thus, future trials are recommended to explore the differences between LA and RA in terms of patient-oriented outcomes.

## Conclusions

In summary, our meta-analysis suggests that RA is advantageous over LA, providing sufficient branchial artery blood flow to guarantee AVF patency whilst increasing brachial artery diameter to avoid thrombosis and several other related complications. Nevertheless, large, head-to-head RCTs with longer follow-up periods are required to support the use of BPB and illustrate the safety differences between RA and LA.

## Data Availability

The datasets used in the analysis was collected by online search, and the datasets analyzed in the current study are available from the corresponding author on reasonable request.

## Abbreviations

AVF: Arteriovenous fistulae
RA: Regional anesthesia
LA: Local anesthesia
GA: General anesthesia
BPB: Brachial plexus block
RCTs: Randomized controlled trials

## References

[1] Riella MC, Roy-Chaudhury P (2013). Vascular access in haemodialysis: strengthening the Achilles’ heel. Nat Rev Nephro.

[2] Lo Monte AI (2011). Comparison between local and regional anesthesia in arteriovenous fistula creation. J Vasc Access.

[3] Sahin L (2011). Ultrasound-guided infraclavicular brachial plexus block enhances postoperative blood flow in arteriovenous fistulas. J Vasc Surg.

[4] Shoshiashvili V, et al. Evaluation of efficacy of regional and local anesthesia techniques in arteriovenous fistula criation for dialysis. Georg Med News. 2014;(236):7–12. 25541817

[5] Meena S (2015). Ultrasound-guided supraclavicular brachial plexus anaesthesia improves arteriovenous fistula flow characteristics in end-stage renal disease patients. South Afr J Anaesth Anal.

[6] Aitken E (2016). Effect of regional versus local anaesthesia on outcome after arteriovenous fistula creation: a randomised controlled trial. Lancet.

[7] Nofal WH (2017). Ultrasound-guided axillary brachial plexus block versus local infiltration anesthesia for arteriovenous fistula creation at the forearm for hemodialysis in patients with chronic renal failure. Saudi J Anaesth.

[8] Mouquet C (1989). Anesthesia for creation of a forearm fistula in patients with endstage renal failure. Anesthesiology.

[9] Solomonson MD, Johnson ME, Ilstrup D (1994). Risk factors in patients having surgery to create an arteriovenous fistula. Anesth Analg.

[10] Malinzak EB, Gan TJ (2009). Regional anesthesia for vascular access surgery. Anesth Analg.

[11] Malovrh M (2003). The role of sonography in the planning of arteriovenous fistulas for hemodialysis. Semin Dial.

[12] Ebert B, Braunschweig R, Reill P (1995). Quantification of variations in arm perfusion after plexus anesthesia with color Doppler sonography. Anaesthesist.

[13] Brimble KS, Rabbat CG, Schiff D, Ingram AJ (2001). The clinical utility of Doppler ultrasound prior to arteriovenous fistula creation. Semin Dial.

[14] Abrahams MS, Aziz MF, Fu RF, Horn JL (2009). Ultrasound guidance compared with electrical neurostimulation for peripheral nerve block: a systematic review and meta analysis of randomized controlled trials. Br J Anaesth.

[15] Rang S (2006). Anaesthesia for chronic renal disease and renal transplantation. EAU-EBU Update Ser.

[16] Capdevila X, Biboulet P, Morau D (2008). How and why to use ultrasound for regional blockade. Acta Anaesthesiol Belg.

[17] Marhofer P, Schrögendorfer K, Koinig H, Kapral S, Weinstabl C, Mayer N (1997). Ultrasound guidance improves sensory block and onset time of three-in-one blocks. Anesth Analg.

[18] Ismail A (2017). Abdelrahman Ibrahim Abushouk.: regional versus local anesthesia for arteriovenous fistula creation in end-stage renal disease: a systematic review and meta-analysis. J Vasc Access.

